# Study on the stability enhancement mechanism of a high-speed axial compressor under paired swirl distortion

**DOI:** 10.1038/s41598-026-56360-5

**Published:** 2026-06-05

**Authors:** Bangqin Cheng, Yuan Li, Zhilong Yin, Yang Yu, Jiajun Liu

**Affiliations:** Air Force Engineering University, Xi’an, 710038 China

**Keywords:** High-speed axial compressor, Swirl distortion, Paired swirl, Stall margin, Peak efficiency, Casing treatment, Stability enhancement mechanism, Energy science and technology, Engineering

## Abstract

Serpentine and buried air intake configurations are widely employed in modern fighter aircraft, which induces significant swirl distortion at the inlet and adversely affects compressor operational stability. This study investigates the instability mechanism and stability enhancement technology for high-speed axial compressors under paired swirl, employing combined numerical simulation and experimental verification. Research indicates that compressor stall is primarily caused by expansion of the tip leakage vortex and flow blockage from the forward migration. Casing treatment efficiently alleviates flow blockage by suppressing the formation and development of the tip leakage vortex, which not only improves the stall margin but also restructuring the radial flow pattern to improve the total pressure ratio, thereby facilitating more stable performance in high-speed compressors. In simulations and experiments, casing treatment increased the stall margin by 52.66% and 50.40%, respectively, while the peak efficiency saw only minor reductions of 0.05 and 0.13 percentage points, respectively.

## Introduction

The demand for stealth performance and super-maneuverability of aircraft in modern air combat has been continuously increasing. Complex configurations, such as serpentine and buried air intakes, are extensively employed in advanced fighter aircraft as they can significantly enhance engine performance^1^. These complex configurations can improve the aircraft’s stealth and aerodynamic performance.However, they affect the uniformity of the engine’s inlet flow field, significantly increasing the probability of swirl distortion at the air intake exit. This situation poses a serious threat to the stability of engine operation,which, in severe cases, may trigger engine surge, presenting risks to flight safety.

In the 1980s, the European Tornado fighter aircraft encountered multiple engine surges during maneuvering flights. This was attributed to an inadequate assessment of the swirl distortion induced by the S-shaped air intake^2^. Likewise, the US Tomahawk cruise missile was also influenced by dynamic swirl distortion, which compelled it to urgently upgrade the throttling algorithm of its control system^3^. Subsequently, research on swirl distortion has gradually attracted attention. Genssler et al.^4^ introduced the first two methodologies for designing swirl distortion generators intended for engine ground testing. Flitcroft et al. and Govardhan et al.^5,6^ utilized similar swirl distortion generators to examine how bulk swirl distortion impacts compressor performance. Pazur and Schmid et al.^7,8^ set up delta wings in a wind tunnel and generated paired swirls of different intensities by changing the angle of attack, and then studied the impact of paired swirls on the performance of the compressor. Sheoran and Bouldin^9,10^ conducted CFD numerical simulations on the designed swirl chambers and obtained several different types of swirl distortion. To establish standardized evaluation systems for swirl distortion, the S-16 Committee formulated and issued the AIR5686 standard^11^, providing a formal basis for associated studies.

With significant progress and innovation in the design methods of swirl distortion generators, researchers have achieved a large number of results. Hoopes et al.^12^ developed a swirl distortion generator using a blade-based approach. Building on this, Hayden et al.^13^ designed High Throughflow StreamVane Swirl Distortion Generators. Tu et al.^14^ developed a variable blade-type swirl distortion generator and examined how swirl distortion affects the performance and operational stability of low-speed compressors. Chen et al.^15^ designed a combined distortion generator of total pressure and paired swirl and employed stereoscopic particle image velocimetry (S-PIV) technology to examine its flow field characteristics and distortion indicators. Zhang et al.^16^ put forward a design approach for a variable camber blade-type swirl distortion generator capable of generating typical paired vortices and overall vortices. Wang et al.^17^ designed a novel type of dynamic distortion simulator that can satisfy the requirements of engine intake matching tests. Lian et al.^18^ investigated how varying swirl inlet conditions influence compressor performance by modifying the mounting angle of the blade-type swirl distortion generator.

With the improvement of computer performance, researchers have been able to draw very fine grids and use CFD technology to conduct full-stage numerical simulations of compressors. As a result, a large number of numerical simulation studies on inlet distortion have been carried out. Hah^19^ conducted an unsteady numerical simulation study on the influence of dynamic total pressure distortion on transonic compressor rotors. Gorrell and Hah^20,21^ employed the LES (Large Eddy Simulation) model to investigate the impact of total pressure distortion on compressor performance. Cousin et al.^22^ used parallel compressor theory to explain the influence of bulk swirl and paired swirl on engine performance. Sheoran^23^ carried out high-precision coupled numerical simulations using a designed cavity-type swirl distortion generator and the ASE120 low-pressure compressor stage. Cheng et al.^24–27^ employed various types of swirl distortion generators to simulate typical swirl distortions with different intensities and carried out systematic studies on how such distortions affect compressor performance. Wang et al.^28^ employed a cavity-based model to study the impact of bulk swirl distortion on the surge characteristics of an axial-flow and centrifugal combined compressor. Xu^29^ established a MACAM-SD computational model capable of accurately predicting the performance and stability boundaries of compressors under swirl intake conditions. Liu^30^ constructed a model to characterize the coupled interaction between inlet distortion and the fan, and subsequently modified the conventional parallel compressor model accordingly. Tian et al.^31^ based on three-dimensional body force model (BFM) calculation program, studied the performance and stability of engine’s boost stage under conditions of swirl distortion, total pressure distortion, and their combined effects. Sun et al.^32^ further explored the distortion transmission characteristics and internal flow field features of the fan using the same approach. Chen et al.^33^ established a definition method for the boundary condition of the offset pair swirl and investigated the coupling effect between the offset pair swirl distortion and the fan. Miao et al.^34^ constructed a comprehensive model integrating the S-bend diffuser section of the inlet duct with the axial-flow compressor rotor, and conducted an in-depth analysis of the mechanisms and characteristics governing swirl distortion formation, along with its effects on compressor performance.

The impact of swirl distortion on compressor stability cannot be overlooked, and it is imperative to conduct in-depth exploration of its mechanism and effective stability enhancement methods. The casing treatment was initially discovered by Hartmann^35^ et al. during the process of conducting venting experiments. In the late 1960s, Oscarson et al.^36^ used four different types of honeycomb casing treatments and conducted experiments on different rotors, all achieving good stability expansion effects. Since then, casing treatment technology has gradually become a core area of research for improving the stability of compressors^37^. Moore and Takatal^38,39^ proposed that the casing should cover the middle area of the rotor and that increasing the depth of the slots would enhance the stability expansion effect. Lee and Greitzer^40,41^ combined the tangential blowing technology with the circumferential slot-type casing treatment, achieving a better stability expansion effect. Based on the above research results, researchers also conducted specialized studies on the specific structural parameters of slot-type and slit-type casings. Moss et al.^42^ studied the influence of the inclination direction on the stability expansion effect of slit-type casings and proved that only when the inclination direction of the slits follows the rotor’s rotation direction will the casing be effective. Moore^43^ conducted research on the influence of four tip clearances ranging from 0.61 to 1.78 mm on casing treatment, proving that an increase in tip clearance has a negative impact on stability expansion. Prince^44^ added a partition in the middle of the treatment slot, but the results showed that the partition had a negative effect on the stability expansion effect. From the 1980s to the 1990s, the main research content was to minimize the efficiency loss caused by casing treatment while ensuring the stability expansion effect. Among them, Wisler and YuQing et al.^45,46^ were representative. They respectively designed oblique groove type casing treatment and arc oblique groove casing treatment, increasing the efficiency by 1–2 percentage points. The casing designed by Wisler slightly reduced the stability expansion effect, while the casing treatment designed by YuQing could achieve a stability expansion effect of over 20%. In recent years, with the advancement of unsteady flow control technology, the understanding of casing treatment mechanisms has become more profound. Yin et al.^47^ developed an adaptive casing treatment technology equipped with annular cavity guide vanes and investigated the influence of different guide vane configurations on the flow field of a subsonic multistage axial-flow compressor. Tong et al.^48^ studied the changes in the stable operating range and performance of an S-CO centrifugal compressor after adopting self-circulating casing treatment. Wang et al.^49^ examined the impact of circumferential slot structure parameters on enhancing compressor stability and determined the optimal parameters. Meanwhile, Yu et al.^50^ demonstrated that the axial casing treatment with an optimized position design has a stronger stability improvement capability and lower efficiency loss. Nevertheless, current studies mainly focus on the effects of total pressure and swirl distortion on compressor operational performance, as well as the stability improvement mechanisms of casing treatment under standard inlet conditions. There remains a dearth of in-depth investigations into how swirl distortion specifically modifies the instability mechanism within the compressor, particularly the distinctive stability enhancement mechanism of casing treatment under swirl distortion conditions. Hence, it is extremely essential to conduct research on the instability mechanism and stability enhancement technology of a high-speed axial compressor under paired swirl distortion conditions.

A specific type of single-stage high-speed axial compressor was selected as the research subject. By integrating full-channel numerical simulations with experimental studies, the effects of paired swirl flow conditions on the flow field instability of compressor were comprehensively examined. Based on this, a casing treatment was designed and research on stability enhancement was conducted. The mechanisms by which casing treatment suppresses flow instability and restructures the flow field under swirl distortion conditions were examined, providing theoretical support for enhancing performance of power systems in modern advanced fighter aircraft.

## Method

### Physical model and calculation method

#### Research object

**Fig. 1:**
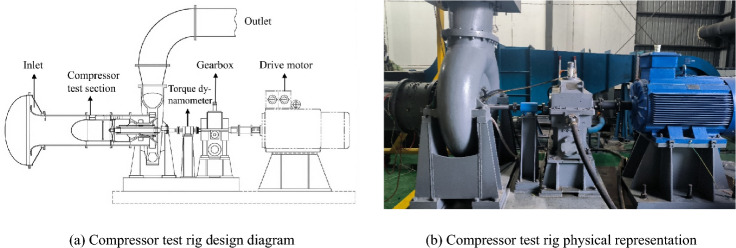
High speed axial compressor test rig.

This paper designated a specific type of single-stage high-speed axial compressor with a design speed of 8000 r/min as the primary research object. Figure 1 depicts the fundamental components of the single-stage axial compressor test rig. The key performance parameters are presented in Table 1.

**Table 1:** Experimental parameters of the single-stage axial compressor.

Parameter	Numerical value
Design speed/(r/min)	8000
Flow rate/(kg/s)	9.20
Total pressure ratio	1.15
Isentropic efficiency/%	89
Hub-to-tip ratio	0.75
Tip inlet Mach number of rotor	0.78
Tip inlet Mach number of stator	0.58
Blade solidity of rotor	0.79
Blade solidity of stator	0.63
Blade installation angle of rotor/°	− 51.94
Blade installation angle of stator/°	34.22
Number of rotor blades	29
Number of stator blades	43
Casing diameter/mm	480

The compressor test rig system primarily comprised a drive motor, a speed increaser, a torque dynamometer, the compressor test section, and the exhaust section. The drive motor had a maximum power of 315 kW and a maximum rotational speed of 3000 r/min. The speed increaser served to adjust the speed ratio, with the maximum speed ratio of 6. The torque dynamometer was employed to monitor the driving power and physical rotational speed in real-time. The compressor test section utilized a mesh air intake hood to simulate the actual flow field, and the exhaust section was furnished with a high-precision servo cylinder to control the throttle valve (with a displacement accuracy of 0.01 mm) for the purpose of achieving precise flow regulation.

**Fig. 2:**
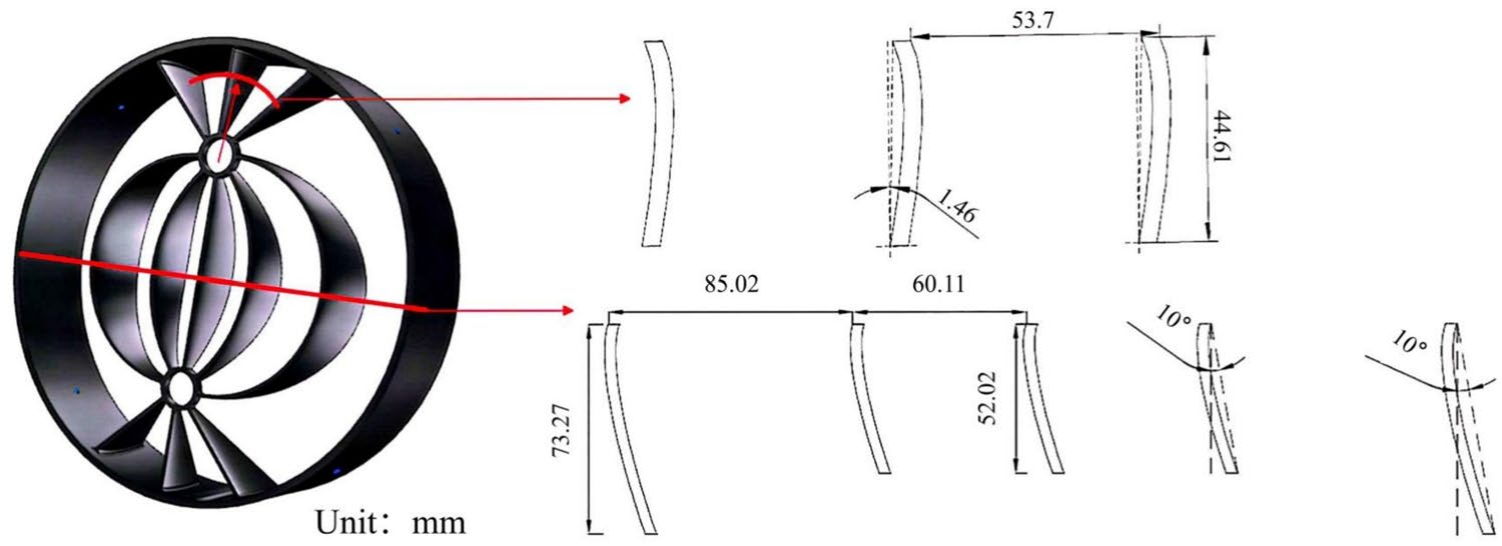
Improved paired swirl distortion generator model.

The swirl distortion generator is a device employed to effectively reproduce the target flow field and simulate the actual inlet conditions of the compressor during its operation. To simulate the paired swirl inlet conditions in actual flight, an enhanced paired swirl distortion generator model was adopted. The model of the distortion generator, with an inner diameter of 480 mm, is depicted in Fig. 2. The distortion generator utilizes the NACA-A4K6 cambered line, featuring a blade profile with a uniform thickness of 4 mm and a blade bend angle of 10°. This distortion generator is a standard paired swirl distortion generator that can generate a pair of vortices of equal size and intensity but opposite directions at the two vortex cores on its sides.

In this paper, the orthogonal experimental design method was adopted for the casing treatment, and 16 numerical tests were carried out for four parameters: slit coverage length *L*_s_ (ranging from 0 to 15 mm), slit width *W* (ranging from 3.0 to 6.5 mm), slit height *H* (ranging from 4 to 12 mm), and the number of slits *N* (ranging from 3 to 5). The preliminary screening results indicated that the parameter combination of *L*_s_ = 15 mm, *W* = 3.6 mm, *H* = 12 mm, and *N* = 116 exhibited the optimal comprehensive performance. Based on this, we further fine-tuned the radial inclination angle of the slit *θ* and the extension distance of the slit upstream beyond the blade leading edge *L*_b_.

**Fig. 3:**
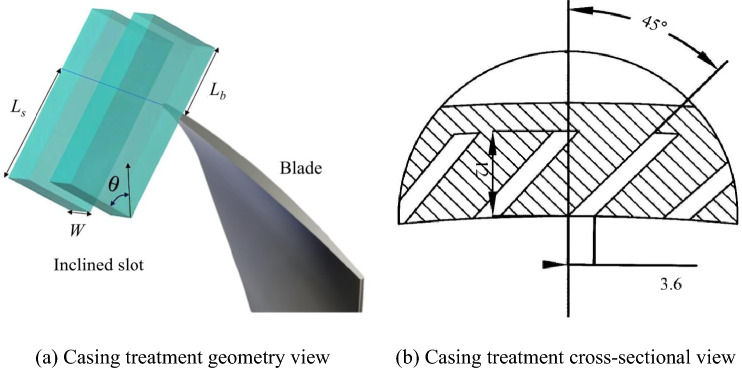
Casing treatment experimental component design.

The structural parameters of the final designed new type casing treatment are as follows: slit width *W* = 3.6 mm, slit coverage length *L*_s_ = 15 mm, the extension distance of the slit upstream beyond the blade leading edge *L*_b_ = 10 mm, the radial inclination angle of the slit *θ* = 45°, slit height *H* = 12 mm, and the number of slits *N* = 116. The design drawing of the casing treatment is presented in Fig. 3.

**Fig. 4:**
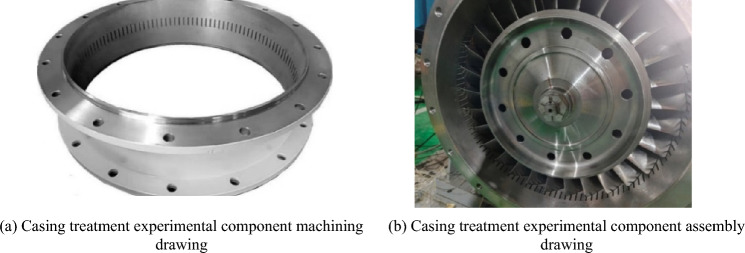
Casing treatment experimental component machining and assembly drawing.

The experimental component of the casing treatment is presented in Fig. 4. During the experiment, only the solid-wall casing of the compressor rotor in the test rig depicted in Fig. 4 was replaced, whereas the other components remained unaltered. This was done to ensure that all variations in the compressor performance were attributable to the influence of the casing treatment.

#### Calculation method

To prevent the circumferential averaging effect of traditional rotor–stator interfaces (e.g., Frozen Rotor, Stage, etc.) from weakening the fine vortex structures upstream, while also taking into account the computational resource cost, this paper employed a coupled numerical strategy of ANSYS CFX and NUMECA to disclose the flow field characteristics of the compressor under the influence of paired swirl. First, the unsteady flow field of the swirl distortion generator was computed based on the ANSYS CFX software. Subsequently, the calculated swirl distortion flow field was utilized as the inlet condition of the compressor, and a full-channel numerical simulation of the high-speed axial compressor was conducted using the NUMECA FINE/Turbo software. For the simulation incorporating the casing treatment, the General Grid Interface (GGI) method was employed to manage the interface between the rotor blade passage and the casing treatment cavity, which allowed the flux transfer between non-matching grids and maintained the conservation of mass, momentum and energy.

**Fig. 5:**
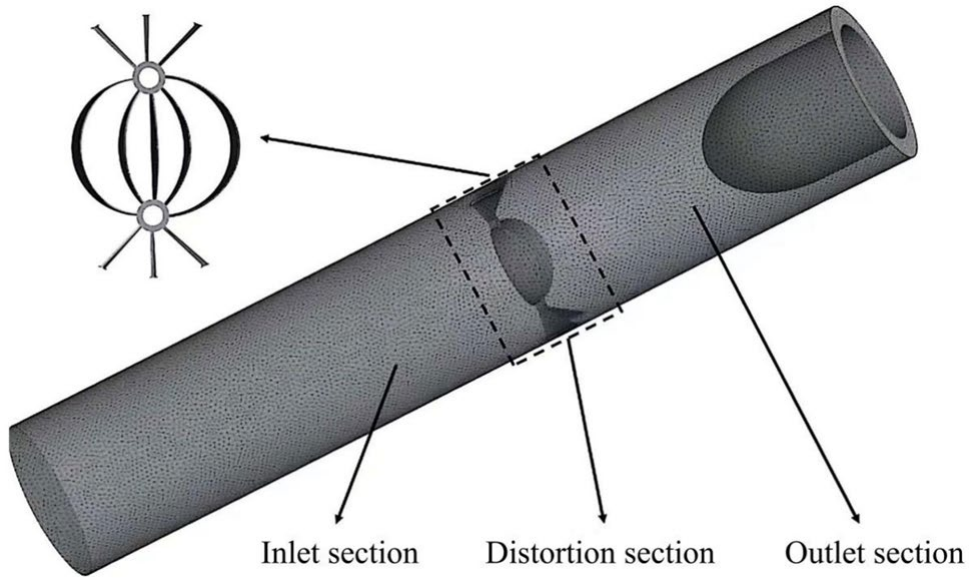
Grid topology of the paired swirl generator.

For the swirl distortion generator part, ANSYS ICEM CFD was employed to generate an unstructured tetrahedral hybrid mesh, as depicted in Fig. 5. The total number of global mesh cells amounted to 1.5 million. As per the standard definition, the minimum orthogonal quality coefficient of the blade section mesh was greater than 0.2. Orthogonal quality is an important indicator for evaluating the deviation of the mesh element from orthogonality, with a value range from 0 to 1. The closer to 1, the better the orthogonality of the mesh, and the higher the calculation accuracy and convergence. The minimum value of 0.2 indicated that the mesh still maintained an acceptable orthogonality level in the most complex geometric positions on the blade surface and near the wall.

The calculation of the swirl distortion generator adopted the SST turbulence model, which has higher accuracy in simulating strong swirls, large curvature walls and free shear flows. The gas medium was treated as an ideal gas, and the wall boundaries were set as adiabatic with no-slip conditions. An axial inlet condition was applied, with the total pressure specified as 101,325 Pa and the total temperature as 288.15 K. The mass flow rate was set at the outlet.

**Fig. 6:**
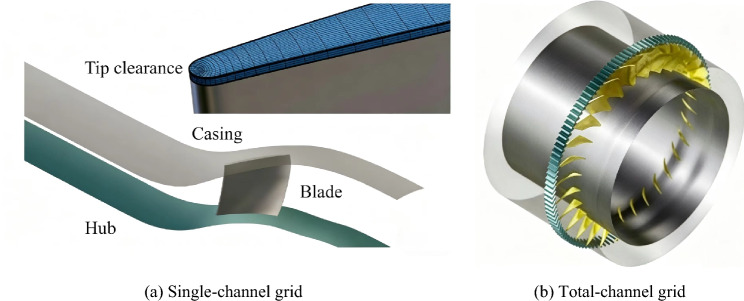
Topological structure of the compressor computational grid.

For the axial compressor part, the structured body-fitted grid of the rotor passage was generated using NUMECA AutoGrid5, whereas the inlet and outlet extension grids were generated within the IGG module, as depicted in Fig. 6. The computational model included the inlet extension section of the compressor to realistically simulate the development of the inlet boundary layer under actual operating conditions. In a single compressor channel, the rotor surface adopted O-type body-fitted grids, with 33 × 85 × 137 grid points, which rotated synchronously with the blades during the calculation. The inlet and outlet sections adopted H-type grids, with 33 × 85 × 33 and 33 × 85 × 25 grid points respectively. All grids were refined near the wall to ensure y^+^ < 2, with each single passage containing approximately 859,000 grid points. The full channel grids were generated by circumferential periodic extension, and each channel was perfectly matched and connected. Full-channel models were employed to simulate the solid-wall casing and the casing treatment, incorporating 19.1 million and 24.9 million grid points, respectively.

In view of the strong adverse pressure gradient and complex vortex structure within the compressor, the k-ε model, which is well adapted to separated flow, was selected for the numerical simulation of the compressor. The gas medium was treated as an ideal gas, and the wall boundaries were set as adiabatic with no-slip conditions. The second-order upwind difference scheme was adopted for spatial discretization, and second-order and fourth-order artificial viscosity were introduced to suppress numerical oscillations. For the simulation under uniform inlet conditions, the inlet was defined as an axial flow with a total pressure of 101,325 Pa and a total temperature of 288.15 K, while the outlet was assigned a static pressure boundary condition. For the swirl inlet condition simulation, the calculated swirl flow field at the corresponding mass flow rate was converted into a Profile file to serve as the inlet boundary condition, while the outlet was assigned a static pressure boundary. During the computation, the compressor’s operating flow rate was varied by adjusting the back pressure to obtain the compressor’s performance characteristic curve. During the computation, the working flow rate of the compressor was changed by adjusting the back pressure to obtain the performance characteristic curve.

To comprehensively analyze the swirl distortion at the air intake exit, the swirl angle distribution at the 0.5D cross-section (AIP) downstream of the swirl distortion generator was obtained through numerical simulation, and supplementary analysis was carried out using velocity vector diagrams and cloud diagrams of swirl angle. The swirl angle α is defined as:

1 $$\alpha = tan^{ - 1} \frac{{U_{\theta } }}{{U_{x} }}$$

Here, *U*_*θ*_ represents the circumferential velocity component at a specific point on the AIP, and *U*_*x*_ denotes the axial velocity component at that same point on the AIP. When observed from the outlet towards the inlet of the AIP, the swirl angle in the counter-clockwise direction is considered positive.

#### Grid independence verification

To conserve computing time and resources while ensuring calculation accuracy, a grid independence verification was conducted on the compressor with casing treatment. Three sets of grids with different densities were partitioned, namely a coarse grid (254,000 cells per channel), a baseline grid (859,000 cells per channel), and a fine grid (2,586,000 cells per channel).

**Fig. 7:**
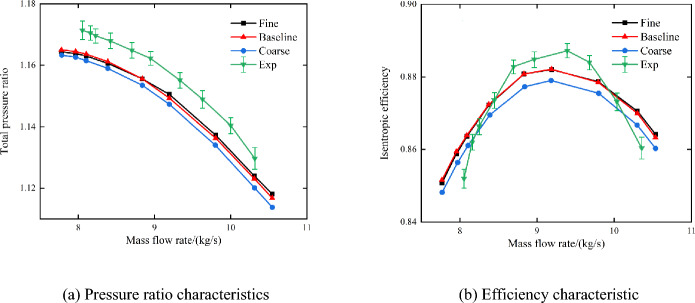
Grid independence verification of the compressor with casing treatment.

The comparison results of the high-speed compressor characteristics simulation experiment at 100% conversion speed are presented in Fig. 7. This figure depicts the comparison between the single-channel numerical simulation results and the experimental results. Through this comparison, it had been found that when the number of single-channel grids reached 859,000 (Baseline grid), further increasing the grid density had minimal impact on the calculation results. Therefore, after comprehensively considering both the calculation accuracy and the calculation speed, it had been ultimately determined to use baseline grid for the solution calculation. Under this grid density, the full-channel model consisted of approximately 24.9 million grid cells in total.

The total pressure ratio and isentropic efficiency derived from the simulation were marginally lower than the experimental outcomes. This can be primarily attributed to the following factors: 1) the numerical dissipation inherent in the finite-volume scheme; 2) the unavoidable errors in the turbulence model; 3) the difficulty in precisely matching the experimental inlet boundary conditions (e.g., turbulence intensity and boundary layer thickness). The grid independence verification indicated that at the chosen grid density (baseline), the calculation error remained relatively stable and can satisfy the research requirements.

### Experimental system and measurement methods

Based on a single-stage high-speed axial compressor test rig with a design speed of 8000 r/min, an investigation was conducted into the instability characteristics of the high-speed axial compressor under paired swirl flow conditions and the effectiveness of casing treatment in enhancing stability. The basic components of the test rig were introduced in the previous text. To acquire the compressor characteristic curve, the measurement system primarily consists of measurement subsystems for total pressure ratio, flow rate, isentropic efficiency, and swirl distortion field.

**Fig. 8:**
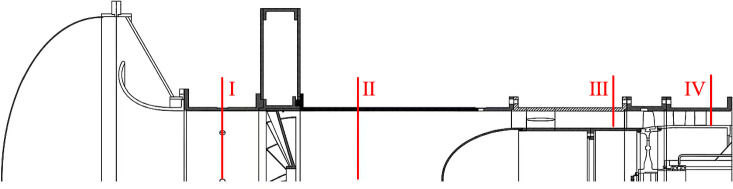
Schematic diagram of the axial distribution of the test section of the experimental article.

The total pressure ratio was determined using total pressure probes, with the measurement system illustrated in Fig. 8. Data were acquired from probes positioned at the compressor inlet (Section III, situated three axial chord lengths upstream of the blade tip leading edge) and at the outlet of the guide vanes (Section IV). At the inlet, three probes were evenly spaced circumferentially at 90° intervals. At the outlet, five measurement points were set along the radial direction, with their arrangement detailed in Table 2. All data were collected through a pressure scanning valve and transmitted to the host computer. After each operating point reached a stable state, 200 sets of data were recorded and averaged to eliminate random errors.

**Table 2:** Distribution of each measuring point along the radial direction.

Measuring point	Measuring point II radius/mm	Measuring point III radius/mm	Measuring point IV radius/mm
1	24	191.00	191.99
2	48	206.35	203.63
3	72	214.90	214.64
4	96	None	225.11
5	120		235.11
6	144		None
7	168		
8	192		
9	216		
10	236		

The flow rate was measured by means of an inlet pitot tube flowmeter. The flow rate value was obtained by measuring the static and total pressure within the flow channel and integrating this information with the atmospheric density. The location of the flowmeter is depicted in Fig. 8 (Section I). The method for data recording and processing was consistent with that of the total pressure ratio.

The isentropic efficiency was measured via the power method. The shaft power comprising torque and speed was measured using a torque sensor, following which the isentropic efficiency of the compressor was calculated. The data recording and processing method remains the same as that of the total pressure ratio.

**Fig. 9:**
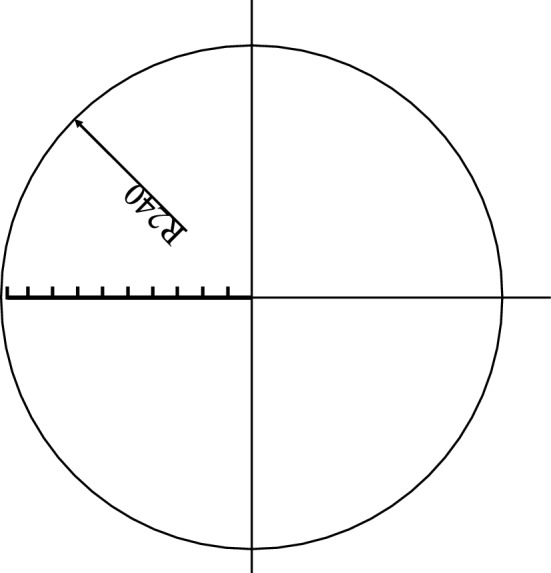
Measurement point II distribution of swirl distortion field.

Swirl distortion field test: A five-hole probe (measurement point Ⅱ) was positioned at a location 0.5D (where D = 480 mm) downstream of the paired swirl distortion generator. During the test, the probe was radially moved within the downstream range of 0 to 240 mm, and the parameters at different radial positions were measured. A total of 10 measurement points were established along the radial direction, and they were uniformly distributed, as depicted in Fig. 9. At the test center point where r = 0 mm, the paired swirl distortion generator remained stationary, meaning that only data at one point was measured. When measuring other measurement points with r > 0 (specific data are provided in Table 2), the paired swirl distortion generator rotates circumferentially by 6° each time. After the flow field stabilizes, the data were recorded via the acquisition system. Based on previous research, the paired swirl distortion fields were all symmetrically distributed. Therefore, the full annular flow field was not tested, and only the flow field distortion distribution within the range of 0 to 180° was examined.

All the aforementioned parameters were transmitted to the upper computer through the acquisition system, and the data processing process was automatically completed by the upper computer program.

## Results and discussion

### Stall mechanism of a high-speed compressor under paired swirl distortion

**Fig. 10:**
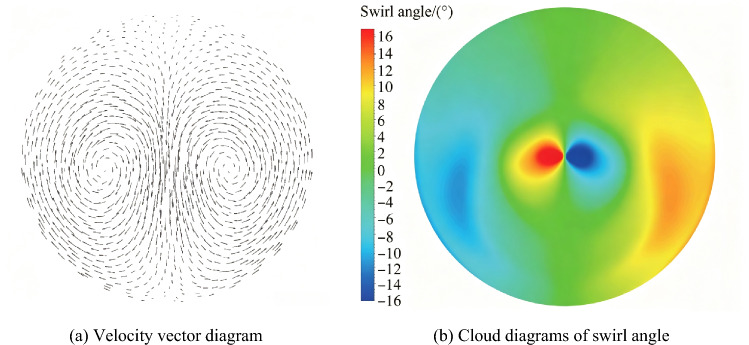
Characteristics of the distorted flow field at the 0.5D cross-section downstream of the swirl distortion generator.

Figure 10 illustrates the characteristics of the distorted flow field at the 0.5D cross-section downstream of the swirl distortion generator. Based on the velocity vector diagram, it is evident that there were two symmetrical and counter-rotating vortex structures on the AIP cross-section. From the cloud diagrams of swirl angle, it can be observed that the absolute value of the swirl angle reached its maximum in the central area of the cross-section, with the maximum positive swirl angle being 16° and the maximum negative swirl angle being − 16°. The swirl angle gradually diminished along the left and right sides outward, while the swirl angles on the upper and lower sides were approximately 0°.

Based on paired swirl inlet conditions, the stall mechanism of a high-speed axial compressor was comprehensively investigated through numerical simulation methods.

**Fig. 11:**
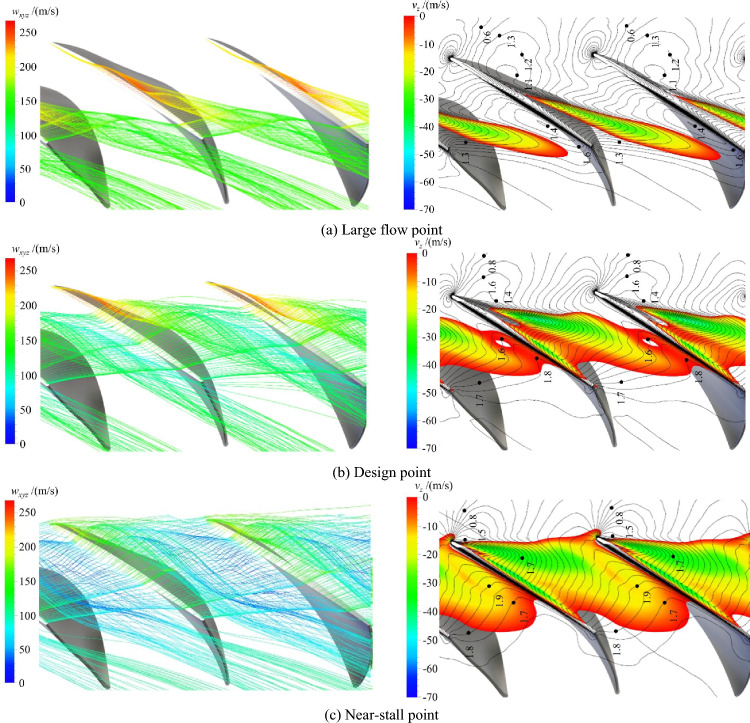
Distribution of TLV and blockage in axial compressor under paired swirl flow condition.

Figure 11 depicts the streamline distribution of the tip leakage flow, the distribution of the 99% blade height axial backflow region (*V*_*z*_ < 0), and the relative static pressure contour distribution under various operating conditions. The low-value area of the relative static pressure contour denoted the trajectory of the TLV, which was marked in the figure with "relative static pressure" as the reference quantity. The relative static pressure is defined as the ratio of the local actual static pressure to the static pressure at the inlet of the computational domain. The analysis showed that, under high-flow conditions, the TLV originates at the mid-chord position of the blade, moves along the suction side, and part of the leakage flow generates secondary leakage flow near the trailing edge of the neighboring blade. A small backflow region existed in the vicinity of the vortex core of the leakage vortex. When the compressor operated at peak-efficiency conditions, the starting position of the TLV advanced, the angle between the vortex core and the blade enlarged, the coverage area of the secondary leakage flow broadened, and the axial backflow area induced by the leakage vortex also expanded. When the compressor as further throttled to a condition approaching stall, the starting position of the TLV advances to the leading edge of the blade tip. The vortex core trajectory becomes more aligned with the circumferential direction, and the leakage vortex undergoes significant expansion. Concurrently, the secondary leakage flow spreads across the full blade span, accompanied by an “overflow” effect appearing near the blade tip’s leading edge. The axial backflow region generated by the TLV almost occupies the entire flow passage, resulting in severe flow blockage at the compressor blade tip. Notably, under the influence of paired inlet swirls, although the pre-swirl provides a slight benefit in postponing stall onset, it modifies the flow structure in the blade tip region, thereby enhancing the interaction between the leakage vortex and the main flow during the vortex development process. The low-energy area of the vortex core expanded at a faster rate, ultimately triggering the same blockage mechanism as that of uniform inlet stall. However, the swirl environment accelerated the development of this process near the stall point.

Therefore, the core impact of paired swirl distortion on compressor stall can be summarized as follows: A slight positive swirl marginally enhanced the stability margin baseline by reducing the equivalent angle of attack. More significantly, the swirl modified the flow field structure and the development environment of the vortex system in the blade tip region. Under near-stall point, this made the expansion effect of the TLV more pronounced, accelerated the accumulation of low-energy fluid, and ultimately induced stall through the same TLV blockage mechanism. Moreover, this process progressed more rapidly as the flow approached the stall point.

### Stability enhancement mechanism of casing treatment in high-speed compressor under paired swirl distortion

Under the paired swirl flow condition, the circumferential non-uniformity at the inlet was notably enhanced. Whether the flow response mechanism within the casing treatment undergoes changes was the crux of its effectiveness in expanding stability. This section concentrates on analyzing how the casing treatment suppresses instability via flow reconstruction under such intricate inlet conditions and deliberates on the similarities and differences in its mechanism of action when compared to uniform incoming flow conditions. The stall margin is defined by the following equation:

2 $$SM = \left[ {\left( {\frac{{\pi_{{{\mathrm{ns}}}} }}{{\pi_{{{\mathrm{ref}}}} }}} \right) \times \left( {\frac{{m_{ref} }}{{m_{ns} }}} \right) - 1} \right] \times 100\%$$

Here, *π* represents the total pressure ratio of the compressor at the corresponding operating point, while *m* represents the mass flow rate of the compressor at the corresponding operating point. The sub-scripts *ns* and *ref* respectively denote the near-stall point and the reference point, where the reference point is typically taken as the peak efficiency point.

**Fig. 12:**
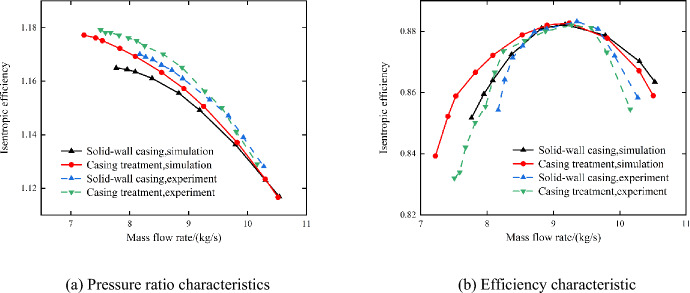
Numerical and experimental results of casing treatment on compressor performance under paired swirl flow condition.

Figure 12 depicts the influence of the casing treatment on the compressor performance under the paired swirl flow condition. The numerical results demonstrated that, under the paired swirl flow condition, the casing treatment elevated the total pressure ratio of the compressor, with the magnitude of improvement progressively increasing as the mass flow rate decreased. The casing treatment diminished the efficiency of the compressor at high flow rates, with the peak efficiency dropping from 88.19% of the prototype to 88.14%, representing a decrease of 0.05 percentage points. At low flow rates, the efficiency ascended, and the degree of ascent gradually increased as the flow rate decreased. Owing to the effect of casing treatment, the compressor can operate at lower flow rates. The stall margin of the compressor increased from 19.90% of the prototype to 30.38%, with relative and absolute changes of 52.66% and 10.48 percentage points, respectively.

Previous research on uniform intake conditions had shown that casing treatment at high flow rates led to a reduction in compressor efficiency. The peak efficiency declined from 88.26% of the original model to 88.24%, representing a decrease of 0.02 percentage points. Conversely, at low flow rates, the efficiency increased, and the magnitude of this increase gradually expanded as the flow rate decreased. Owing to the casing treatment, the compressor was able to operate stably at lower flow rates. The stall margin of the compressor increased from 21.38% of the original model to 30.06%, with relative and absolute changes of 40.60% and 8.68 percentage points, respectively. The stabilizing effect under the paired swirl flow condition is significantly superior to the improved value under the uniform paired swirl condition.

**Fig. 13:**
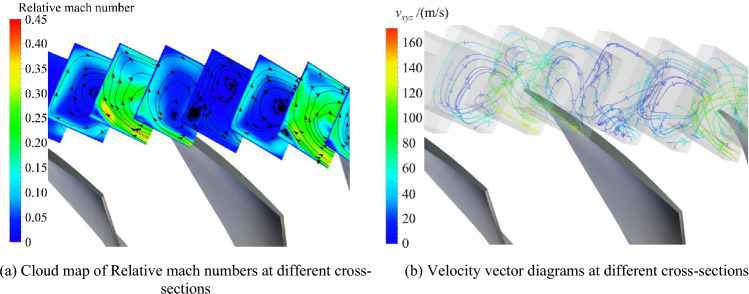
Flow distribution in the casing treatment at the near-stall point of the compressor under paired swirl flow condition.

Figure 13 depicts the velocity distribution and streamline distribution within the casing treatment, in which the Mach number contour was employed to identify high-and low-pressure regions. The area with a low Mach number (blue) corresponded to the high-pressure area, and the area with a high Mach number (yellow) corresponded to the low-pressure area. Owing to the pressure difference between the suction and pressure surfaces of the blades, a flow circulation from the downstream to the upstream was established within the casing treatment slot. A "suction-jet" type of flow exchange was formed between the slot and the compressor channel. The analysis indicates that there was evident circumferential non-uniformity in the flow within the casing treatment. At the slot location aligned with the high-pressure region on the pressure side of the blade leading edge, the suction effect was strongest; in contrast, at positions corresponding to the low-pressure region on the blade suction surface or near the channel midsection, the jet effect became more dominant. This circumferential non-uniform "suction-jet" mode effectively regulated the flow in accordance with the instantaneous pressure distribution within the blade channel, which was the crux of its efficient operation under swirl distortion conditions. The performance enhancement of the compressor achieved through casing treatment was directly attributed to the formation of reverse flow circulation within the slot, moving from downstream to upstream regions.

**Fig. 14:**
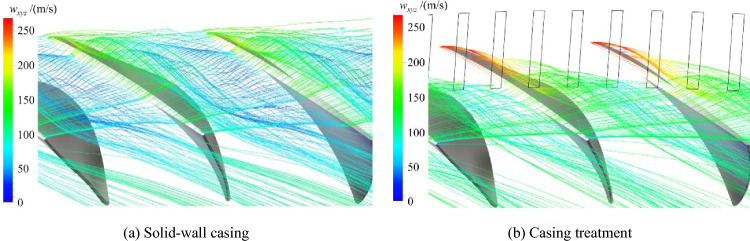
TLV distribution of axial compressor near stall point under paired swirl flow conditions.

**Fig. 15:**
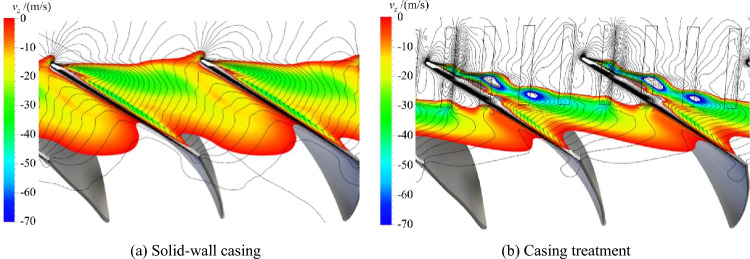
Distribution of tip blockage at the near-stall point of axial compressor under paired swirl flow conditions.

Figure 14 depicts the streamline distribution of the tip leakage flow of the compressor under the near-stall point (corresponding to the minimum flow point of the solid-wall casing) under the paired swirl flow condition, and Fig. 15 presents the corresponding tip blockage and relative static pressure contour distribution. It can be clearly seen that the TLV was significantly weakened when the casing treatment was applied. Its starting position shifted downstream, the angle between the vortex core and the blade diminishes, and no large-scale expansion occurred during its downstream development, leading to a more regular shape. Simultaneously, the tip leakage flow no longer entered the adjacent channel but “overflows”. As a result, the substantial reduction in flow passage blockage caused by the TLV was the primary reason for the increase in the stall margin of the compressor due to the casing treatment. Moreover, it effectively mitigated the enhanced expansion of the TLV caused by swirl distortion, promoting a more stable development state.

**Fig. 16:**
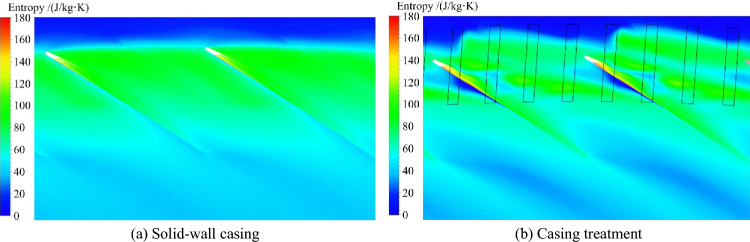
Entropy distribution of 99% blade height at the near-stall point of axial compressor under paired swirl flow conditions.

Figure 16 depicts the entropy distribution at 99% of the blade height for both the solid-wall casing and the casing treatment, operating under identical conditions set at the near-stall point of the solid-wall configuration. For the solid-wall casing, the tip leakage vortex (TLV) spanned the full extent of the flow passage, forcing the leakage flow to closely follow the leading edge of the blade. Such flow behavior led to substantial losses in the blade channel. Implementing casing treatment altered the spatial distribution of these losses within the passage. Although there was a mixing loss caused by the suction and jet effects of the slot, the overall flow loss within the blade channel remains relatively unchanged. This suggested that the slight decrease in efficiency caused by the casing treatment primarily stemmed from the mixing within the slot cavity rather than the degradation of the main channel flow.

**Fig. 17:**
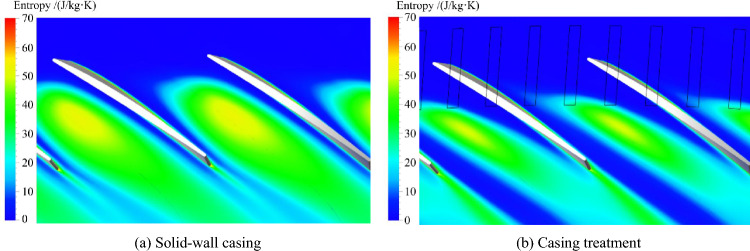
Entropy distribution of 90% blade height at the near-stall point of axial compressor under paired swirl flow conditions.

Figure 17 depicts the entropy distribution at 90% of the blade height for both the solid-wall casing and the casing treatment, under identical operating conditions set at the near-stall point of the solid-wall configuration. It could be observed that the casing treatment significantly reduced the size of the high-entropy region and lowers the peak entropy value within the blade passage. This reduction was attributed to the effective suppression of the TLV, which markedly contracted its radial influence zone. Consequently, the casing treatment mitigated the flow loss in the compressor blade passage by suppressing the TLV. This accounted for the efficiency improvement under low-flow conditions and also suggested that its enhancement effect on the main-channel flow was more pronounced in the medium and low blade height regions.

**Fig. 18:**
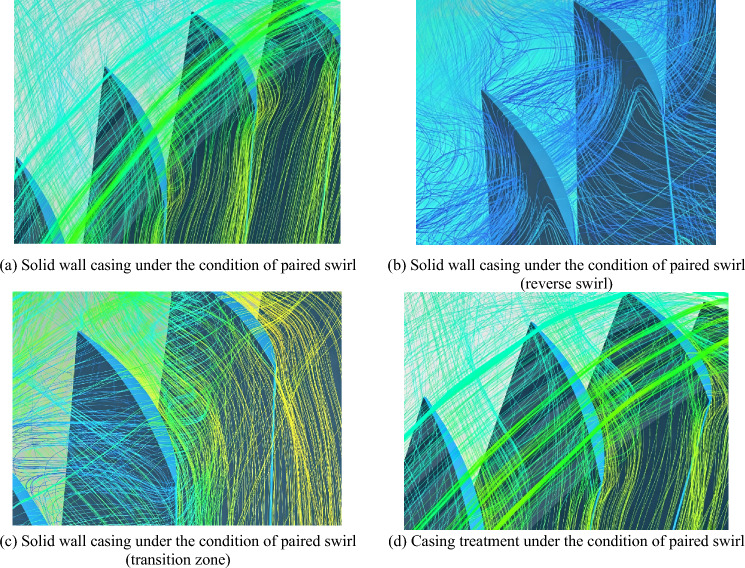
Diagram of blade tip leakage streamline.

Figure 18a–c show the leakage streamline diagrams of the compressor at the tip under paired swirl inlet conditions when it just entered the stall state. Figure 18d presents the leakage streamline diagram of the compressor at the tip at the same flow rate. Figure 18a displays the streamline diagrams in all blade channels of the compressor under the influence of paired swirl. It can be seen from the figure that when a large area of low-speed zone is induced in some regions, causing stall, other regions have not yet stalled. Figure 18b and c magnify the tip channel, showing the area affected by the reverse swirl and the transition area between the area affected by the reverse swirl and the forward swirl. It can be seen from the figure that at this flow rate, the area of the low-speed zone formed after the leakage vortex breaks in the tip channel is significantly larger than that at the near-stall condition, covering the entire tip channel, which severely hinders the fluid flow and causes local stall in the compressor, eventually leading to the stall of the entire compressor. At the same time, it can be found from Fig. 18c that some leakage flow enters the next blade channel, causing the tip clearance leakage flow in this area to roll up, which has a negative impact on the flow in this area. It can be inferred that as the flow rate further decreases, the stall area of the compressor will continue to expand. Figure 18d shows the streamline diagram in the blade channel after the casing treatment. At this flow rate point, the effect of the casing treatment is more obvious. By comparing this figure with Fig. 18a–c, it is found that there are almost no leakage vortices in the tip channel, and most of the fluid flows along the suction surface of the compressor blade into the downstream of the tip channel, while the remaining leakage flow is sucked into the slot casing, reducing the mixing of the tip clearance leakage flow at the leading edge with the mainstream, alleviating the negative impact caused by the paired swirl, and expanding the stable operating margin of the compressor.

**Fig. 19:**
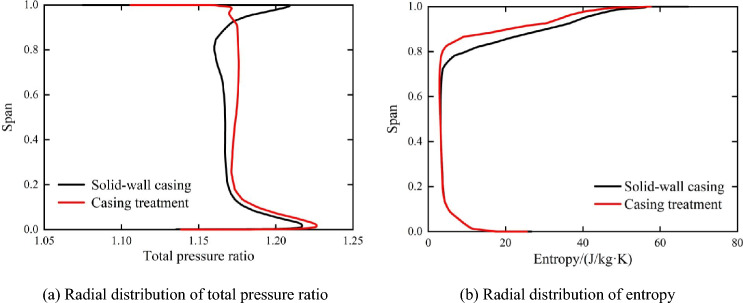
Radial distribution of aerodynamic parameters of axial compressor at the near-stall point under paired swirl flow condition.

Figure 19 depicts the radial profiles of total pressure ratio and entropy for both the solid-wall casing and the casing treatment configuration, where the values represent circumferential averages across all passages. The casing treatment modified the radial distribution of the total pressure ratio compressor. Specifically, the total pressure ratio decreased above 90% span near the blade tip, while it increased in regions below this height. As a result, the casing treatment alleviated the load on the compressor tip and augments the load on the blades in other blade height ranges, thus increasing the overall total pressure ratio of the compressor. Analysis of the radial entropy distribution reveals that the entropy levels near the casing wall remained largely unchanged with the application of casing treatment. Along the radial direction, the entropy value decreased significantly up to around 73% blade height and remained nearly constant below this blade height. This radial flow reconstruction phenomenon clearly demonstrated that the casing treatment effectively unloaded the tip region by extracting the low-momentum fluid at the tip and re-injecting it into the mainstream, compelling the blades in the medium and low blade height regions to bear more load, thereby enhancing the work capacity of compressor and overall total pressure ratio. This represented one of the core aerodynamic mechanisms for its stability expansion. The presence of swirl distortion did not impede the effective operation of this reconstruction mechanism.

### Analysis of stability enhancement effect of casing treatment on high-speed compressor under paired swirl distortion

Figure 12 depicts the effect of casing treatment on compressor performance under paired swirl flow conditions. The experimental results indicate that, across most operating conditions, casing treatment improves the total pressure ratio, with greater improvement observed as the mass flow rate diminishes. At high flow rate conditions, a slight decline in both pressure ratio and efficiency was observed; specifically, the peak efficiency decreased from 88.33% for the baseline configuration to 88.20%, corresponding to a reduction of 0.13 percentage points. In contrast, at low flow rate conditions, the efficiency improved, enabling the compressor to operate under even lower flow rate conditions. The stall margin of the compressor was increased from 16.25% of the original model to 24.44% by the casing treatment, with relative and absolute increases of 50.40% and 8.19 percentage points, respectively.

Previous research on uniform intake conditions had shown that casing treatment at high flow rates led to a reduction in compressor efficiency. The peak efficiency declined from 88.56% of the original model to 88.38%, representing a decrease of 0.18 percentage points. Conversely, at low flow rates, the efficiency increased, and the magnitude of this increase gradually expanded as the flow rate decreased. Owing to the casing treatment, the compressor was able to operate stably at lower flow rates. The stall margin of the compressor increased from 17.86% of the original model to 24.77%, with relative and absolute changes of 38.69% and 6.91 percentage points, respectively. The stabilizing effect under the paired swirl flow condition is significantly superior to the improved value under the uniform paired swirl condition.

The experimental data and simulation results presented in Fig. 12 show a consistent trend, confirming the reliability of the simulation in capturing the overall flow behavior. Nevertheless, disparities existed in the magnitude of change. The experimental results indicated a somewhat larger efficiency loss and a higher margin improvement. The primary reasons were that the numerical model was a simplified one, failing to account for minor discrepancies in boundary conditions. Simultaneously, there were certain measurement errors in the experiment. However, both sets of results confirmed the substantial stabilizing effect of the casing treatment under real swirl distortion conditions.

## Conclusions

The research findings are presented as follows:

1. Under the paired swirl distortion inlet condition, the stall mechanism of the high-speed axial compressor can be summarized as follows: Compressor stall is primarily governed by the forward movement and expansion of the TLV, which leads to the blockage of the flow passage. Although the swirl environment marginally enhances the margin baseline by reducing the equivalent attack angle, it predominantly accelerates the expansion and accumulation of the low-energy fluid of the TLV under near-stall point, ultimately inducing instability via the blockage mechanism.
2. The casing treatment designed in this paper effectively inhibited the generation and propagation of the TLV under swirl distortion conditions via a circumferential non-uniform "suction-jet" flow cycle. This resulted in a rearward shift of the leakage vortex initiation location and stabilization of its core trajectory, thereby effectively mitigating flow passage blockage and significantly enhancing the stall margin. More crucially, the casing treatment mitigated the aerodynamic load in the tip region by extracting low-energy fluid from the tip and re-injecting it into the mainstream. It also reconstructed the radial distribution of the flow, while augmenting the load on the blades in the medium and low blade height range. This kept the efficiency essentially constant while increasing the total pressure ratio, offering an effective stabilization method for high-speed compressors under swirl distortion conditions.
3. Both numerical and experimental investigations confirmed that under the paired swirl flow condition, the casing treatment could effectively mitigated the adverse impacts of the swirl environment on the TLV, thereby significantly enhancing the stability of the compressor. The stall margin experienced a relative increase of 52.66% (numerical)/50.40% (experimental), with an absolute increase of 10.48 percentage points (numerical)/8.19percentage points (experimental). The peak efficiency only exhibited a slight decrease of 0.05 percentage points (numerical)/0.13 percentage points (experimental). Under uniform intake conditions, the stall margin experienced a relative increase of 40.60% (numerical)/38.69% (experimental), with an absolute increase of 8.68 percentage points (numerical)/6.91 percentage points (experimental). The peak efficiency only exhibited a slight decrease of 0.02 percentage points (numerical)/0.18 percentage points (experimental). The stabilizing effect under the paired swirl flow condition is significantly superior to the improved value under the uniform paired swirl condition, providing an effective technical approach for advanced fighter engine to cope with complex intake conditions.

## Data Availability

The datasets used during the current study are available from the corresponding author on reasonable request.
